# Evaluation of the Patients with Recurrent Angina After Coronary Artery Bypass Grafting

**DOI:** 10.21470/1678-9741-2023-0303

**Published:** 2024-05-13

**Authors:** Salih Salihi, Halil İbrahim Erkengel, Fatih Toptan, Bilhan Özalp, Hakan Saçlı, İbrahim Kara

**Affiliations:** 1 Department of Cardiovascular Surgery, Medicine Faculty, Sakarya University, Sakarya, Turkey; 2 Department of Cardiovascular Surgery, Sakarya Training and Research Hospital, Sakarya, Turkey; 3 Department of Anesthesiology and Reanimation, Sakarya Training and Research Hospital, Sakarya, Turkey

**Keywords:** Coronary Artery Bypass, Angina Pectoris, Stroke Volume, Percutaneous Coronary Intervention, Vascular Graft Occlusion

## Abstract

**Introduction:**

In this study, we aimed to evaluate the most common causes of recurrent angina after coronary artery bypass grafting (CABG) and our treatment approaches applied in these patients.

**Methods:**

We included all patients who underwent CABG, with or without percutaneous coronary intervention after CABG, at our hospital from September 2013 to December 2019. Patients were divided into two groups according to the time of onset of anginal pain after CABG. Forty-five patients (58.16 ± 8.78 years) had recurrent angina in the first postoperative year after CABG and were specified as group I (early recurrence). Group II (late recurrence) comprised 82 patients (58.05 ± 8.95 years) with angina after the first year of CABG.

**Results:**

The mean preoperative left ventricular ejection fraction was 53.22 ± 8.87% in group I, and 54.7 ± 8.58% in group II (P=0.38). No significant difference was registered between groups I and II regarding preoperative angiographic findings (P>0.05). Failed grafts were found in 27.7% (n=28/101) of the grafts in group I as compared to 26.8% (n=51/190) in group II (P>0.05). Twenty-four (53.3%) patients were treated medically in group I, compared with 54 (65.8%) patients in group II (P=0.098). There was a need for intervention in 46.6% (n=21) of group I patients, and in 34.1% (n=28) of group II patients.

**Conclusion:**

Recurrent angina is a complaint that should not be neglected because most of the patients with recurrent angina are diagnosed with either native coronary or graft pathology in coronary angiography performed.

## INTRODUCTION

**Table:** 

Abbreviations, Acronyms & Symbols				
AF	= Atrial fibrillation		LAD	= Left anterior descending coronary artery
CABG	= Coronary artery bypass grafting		LIMA	= Left internal mammary artery
CAD	= Coronary artery disease		LMCA	= Left main coronary artery
CAG	= Coronary angiography		LVEF	= Left ventricular ejection fraction
CPB	= Cardiopulmonary bypass		NSTEMI	= Non-ST-elevation myocardial infarction
CX	= Circumflex artery		OPCAB	= Off-pump coronary artery bypass surgery
CX-IM	= Circumflex intermedial artery		PCI	= Percutaneous coronary intervention
CX-OM1	= Circumflex first obtuse marginal artery		RCA	= Right coronary artery
CX-OM2	= Circumflex second obtuse marginal artery		RDP	= Right descending posterior artery
IMA	= Internal mammary artery		SD	= Standard deviation
IR	= Incomplete revascularization		STEMI	= ST-elevation myocardial infarction
ITA	= Internal thoracic artery		SVGs	= Saphenous vein grafts

Coronary artery bypass grafting (CABG) is the recommended treatment for coronary artery disease (CAD) involving left main coronary artery or multiple vessels disease, with a survival benefit compared to percutaneous coronary intervention (PCI)^[1,2]^. Nevertheless, recurrent angina after CABG is really demoralizing because this complaint was the most frequent indication for coronary artery bypass surgery. However, it is frequently known that coronary revascularization procedures do not guarantee complete relief of recurrent angina after the procedure^[3]^. Recurrence of angina in the first year has been reported in 20-30% of patients after successful CABG^[4]^, and it is usually due to a technical problem with a graft. Late recurrent angina can occur with the development of stenosis in a bypass graft or with progression of atherosclerosis in non-bypassed vessels. Failure of the saphenous vein grafts (SVGs) usually occurs without symptoms and does not seem to affect cardiovascular events and mortality after CABG^[5]^. In this study, we aimed to evaluate the most common causes of recurrent angina after CABG and our treatment approaches applied in these patients.

## METHODS

### Patients

From September 2013 to December 2019, 1,183 patients underwent isolated CABG in our institution. We included all patients who had coronary angiography (CAG) with or without PCI after CABG at our hospital in the same period. CAG was performed in a total of 127 patients with respect to standard angiography indications by interventional cardiologists. Patients were divided into two groups according to the time of onset of anginal pain after CABG.

Forty-five patients (10 women, aged 58.16 ± 8.78 years) had recurrent angina in the first postoperative year after CABG and were specified as group I (early recurrence). Group II (late recurrence) comprised 82 patients (17 women, aged 58.05 ± 8.95 years) with angina after the first year of CABG.

CABG with concomitant cardiac procedures, such as valve replacement or repair, aortic replacement, or left ventricular aneurysmectomy, were excluded from this study.

### Data Collection

The study was conducted in accordance with the principles of Helsinki Declaration and was approved by the ethics committee of Sakarya University, Medicine Faculty (approval E-71522473-050.01.04-113296-58, date: 04/03/2022). All preoperative, operative, and postoperative data were reviewed from electronic medical records for each patient.

### Surgical Methods

Standard anesthetic technique was used during induction (fentanyl, midazolam, and pancuronium) followed by the maintenance of isoflurane and propofol. All operations were performed via median sternotomy. The internal thoracic artery (ITA) and SVG were prepared if necessary. Surgical revascularization was performed under cardiopulmonary bypass (CPB) (except for five patients). CPB was established via standard aortic arterial and two-stage venous cannulation. Antegrade cardioplegia delivery cannulas were inserted into the aortic root. In selected patients (left main lesions and acute coronary syndromes), the retrograde cardioplegia cannulas were inserted into the coronary sinus in addition to antegrade cannulas. Diastolic arrest was maintained by delivery of intermittent, moderately hypothermic blood cardioplegia in all patients. Body temperature was maintained between 28°C and 30°C during CPB. Distal anastomoses were performed under aortic cross-clamping while proximal anastomoses were performed with side clamping during rewarming. ITA was routinely applied for left anterior descending artery revascularization, and SVG was anastomosed to other target vessels. Before removal of cross-clamp, a last cardioplegic solution (hot-shot) at 37°C was delivered.

### Statistical Analysis

Data analysis was performed using IBM Corp. Released 2017, IBM SPSS Statistics for Windows, Version 25.0, Armonk, NY: IBM Corp. The variables were investigated using visual (histograms, probability plot) and analytical (Kolmogorov-Smirnov/Shapiro-Wilk’s test) methods to determine whether they are normally distributed. The continuous variables were expressed as mean and standard deviation or as median and interquartile range, depending on the normality of their distribution. The Pearson’s chi-square test (or Fisher’s exact test, where applicable) was used to compare discrete variables, while the independent *t*-test was used for continuous variables between the groups. The statistically significant two tailed *P*-value was considered as < 0.05.

## RESULTS

One hundred and twenty-seven patients were enrolled in the present study according to the inclusion criteria. A total of 45 patients were in group I (22.2% female, mean age 58.16 ± 8.78 years), and 82 patients were in group II (20.7% female, mean age 58.05 ± 8.95 years). Baseline characteristics of all patients are summarized in Table 1.

**Table 1:** Demographic characteristics of all patients.

Characteristics	Group I	Group II	*P*-value
	(n=45)	(n=82)	
Age, years (mean ± SD)	58.16 ± 8.78	58.05 ± 8.95	0.74
Female sex, n (%)	10 (22.2%)	17 (20.7%)	0.84
Associated diseases, n (%)			
Hypertension	39 (86.6%)	68 (82.9%)	0.58
Diabetes mellitus	22 (48.9%)	55 (67.1%)	0.045
Hyperlipidemia	25 (55.5%)	39 (47.6%)	0.39
Cigarette smoking	21 (46.6%)	29 (35.3%)	0.21
Peripheral vascular disease	5 (11.1%)	5 (6.1%)	0.31
Carotis artery disease	8 (17.8%)	8 (9.7%)	0.19
Chronic renal failure^*^	3 (6.7%)	3 (3.6%)	0.44
Preoperative AF, n (%)	0	1 (1.2%)	0.46
Echocardiographic findings			
LVEF (%)	53.22 ± 8.87	54.7 ± 8.58	0.38
Mitral regurgitation, n (%)			0.77
Mild	23 (51.1%)	40 (48.8%)	
Moderate	2 (4.4%)	2 (2.4%)	
Angiographic findings			
LMCA stenosis	4 (8.9%)	16 (19.5%)	0.11
LAD stenosis	45 (100%)	79 (96.3%)	0.19
Diagonal stenosis	5 (11.1%)	7 (8.5%)	0.63
Circumflex stenosis	25 (55.5%)	52 (63.4%)	0.38
RCA stenosis	30 (66.7%)	44 (53.6)	0.15

Data are presented as mean value ± SD or number of patients (percentage)

AF=atrial fibrillation; LAD=left anterior descending coronary artery; LMCA=left main coronary artery; LVEF=left ventricular ejection fraction; RCA=right coronary artery; SD=standard deviation

^*^Creatinine level of > 2 mg/dl

There were no significant differences between the groups with respect to hypertension, hyperlipidemia, cigarette smoking, chronic renal failure, and cerebrovascular disease (*P*>0.05). Diabetes mellitus was seen in 77 patients (group I: 48.9% [n=22]; group II: 67.1% [n=55], *P*=0.045). The mean preoperative left ventricular ejection fraction (LVEF) was 53.22 ± 8.87% in group I, and 54.7 ± 8.58% in group II (*P*=0.38). No significant difference was registered between groups I and II regarding preoperative angiographic findings (*P*>0.05).

Most of the operations (n=122) were performed under CPB, except for five patients in group II (*P*=0.09). Operative data of all patients are summarized in Table 2. Left internal mammary artery was harvested in 122 patients and was not used for left anterior descending coronary artery anastomosis in five patients due to poor flow after harvesting. Mean numbers of grafts of both groups were 2.24 ± 0.8 and 2.33 ± 0.97 in groups I and II, respectively (*P*=0.167). There were no significant differences between the groups with respect to coronary anastomosis sites, coronary endarterectomy, coronary artery quality, and coronary artery diameter (*P*>0.05). Complete revascularization was not achieved in some patients (n=19) because there was no suitable coronary artery for bypass grafting. Incomplete revascularization (IR) of both groups was declared in eight (17.7%) and 11 (13.4%) patients in groups I and II, respectively (*P*=0.51).

**Table 2:** Perioperative parameters.

Variables	Group I	Group II	*P*-value
	(n=45)	(n=82)	
Surgical technique, n (%)			
OPCAB	0	5 (6.1%)	0.09
Grafts, n (%)			
LIMA	42 (93.3%)	75 (91.4%)	0.71
Venous graft	37 (82.2%)	65 (79.3%)	0.69
Number of grafts	2.24 ± 0.8	2.33 ± 0.97	0.167
Coronary anastomosis site, n (%)			
LAD	45 (100%)	82 (100%)	
LIMA	42 (93.3%)	75 (91.4%)	0.71
Venous graft	3 (6.7%)	7 (8.5%)	0.71
Diagonal	3 (6.7%)	9 (11%)	0.43
Circumflex	27 (60%)	48 (58.5%)	0.87
RCA	16 (35.5%)	16 (19.5%)	0.04
RDP	10 (22.2%)	27 (32.9%)	0.20
Coronary artery diameter			
< 1 mm	9 (20%)	17 (20.7%)	0.92
Coronary artery quality			
With heavy plaque	14 (31.1%)	28 (34.1%)	0.73
Coronary endarterectomy	1 (2.2%)	7 (8.5%)	0.15

Data are presented as mean ± standard deviation or number of patients and percentage

LAD=left anterior descending coronary artery; LIMA=left internal mammary artery; OPCAB=off-pump coronary artery bypass surgery; RCA=right coronary artery; RDP=right descending posterior artery

### Indications for Coronary Angiography and Treatment Approaches

The average time between the cardiac operation and CAG was 8.31 ± 3.63 months in group I and 48.87 ± 21.14 in group II. The distribution of indications for CAG was similar in both groups. The primary indication for undergoing CAG included 10 (22.2%) patients with ST-elevation myocardial infarction in group I and nine (10.9%) patients in group II (*P*=0.235). Angiographic findings were summarized in Table 3. There was no significant difference between groups with respect to new native vessel stenosis (*P*>0.05). Failed grafts were found in 27.7% (n=28/101) of the grafts in group I as compared to 26.8% (n=51/190) in group II (*P*>0.05). Twenty-four (53.3%) patients were treated medically in group I, compared with 54 (65.8%) patients in group II (*P*=0.098). There was a need for intervention in 46.6% (n=21) of group I patients, and 34.1% (n=28) of group II patients. The distribution of treatment approaches applied in all patients according to the results of CAG is shown in Table 4. Comparing group I with group II, intervention of graft failure (six [13.3%] and three [3.6%], respectively) was higher in group I, but there was no statistically significant difference (*P*=0.098).

**Table 3:** Postoperative angiographic findings.

Variables	Group I	Group II	*P*-value
	(n=45)	(n=82)	
Time of coronary angiography, months	8.31 ± 3.63	48.87 ± 21.14	0.000
Indication for postoperative angiography, n (%)			0.236
Stabile angina pectoris	19 (42.2%)	40 (48.7%)	
NSTEMI	16 (35.5%)	33 (40.2%)	
STEMI	10 (22.2%)	9 (10.9%)	0.235
Anterior	1 (2.2%)	1 (1.2%)	
Inferior	9 (20%)	8 (9.7%)	
Angiographic findings			
No coronary or graft pathology, n (%)	11 (24.4%)	25 (30.5%)	0.84
New native vessel stenosis, n (%)			
LMCA stenosis	2 (4.4%)	1 (1.2%)	0.286
LAD stenosis	0 (0%)	3 (3.6%)	0.266
Diagonal stenosis	6 (13.3%)	6 (7.3%)	0.268
Circumflex stenosis	6 (13.3%)	7 (8.5%)	0.394
RCA stenosis	4 (8.9%)	15 (18.3%)	0.155
Graft pathology, graft (%)			
LIMA-LAD anastomosis occlusion	6/42	8/75	0.538
LAD venous graft occlusion	2/3	2/7	0.614
Diagonal venous graft occlusion	1/3	3/9	0.55
CX-IM venous graft occlusion	0/7	0/1	0
CX-OM1 venous graft occlusion	3/5	8/21	0.554
CX-OM2 venous graft occlusion	7/15	12/34	0.889
RCA venous graft occlusion	5/16	4/16	0.19
RDP venous graft occlusion	4/10	14/27	0.206

Data are presented as mean ± standard deviation or number and percentage

CX-IM=circumflex intermedial artery; CX-OM1=circumflex first obtuse marginal artery; CX-OM2=circumflex second obtuse marginal artery; LAD=left anterior descending coronary artery; LIMA=left internal mammary artery; LMCA=left main coronary artery; RCA=right coronary artery; RDP=right descending posterior artery; NSTEMI=non-ST-elevation myocardial infarction; STEMI=ST-elevation myocardial infarction

**Table 4:** Treatment approaches of all patients after coronary angiography.

Variables	Group I	Group II	*P*-value
	(n=45)	(n=82)	
Maximum medical treatment, n (%)	24 (53.3%)	54 (65.8%)	0.166
No coronary or graft pathology	11 (24.4%)	25 (30.5%)	
New native vessel stenosis	3 (6.7%)	3 (3.6%)	
Graft pathology	10 (22.2%)	26 (31.7%)	
Intervention of native coronary artery, n (%)	15 (33.3%)	25 (30.5%)	0.741
LAD	2 (4.4%)	1(1.2%)	
Diagonal	1 (2.2%)	2 (2.4%)	
Circumflex	2 (4.4%)	7 (8.5%)	
RCA	10 (22.2%)	15 (18.3%)	
Intervention of graft, n (%)	6 (13.3%)	3 (3.6%)	0.067
LIMA-LAD	2 (4.4%)	1 (1.2%)	
Aorta-LAD	2 (4.4%)	0	
Aorta-CX	1 (2.2%)	1 (1.2%)	
Aorta-RCA	1 (2.2%)	1 (1.2%)	

Data are presented as number of patients (percentage)

CX=circumflex artery; LAD=left anterior descending coronary artery; LIMA=left internal mammary artery; RCA=right coronary artery

## DISCUSSION

In this study, we aimed to evaluate the most common causes of recurrent angina after CABG and our treatment approaches applied in these patients. Comparison of the two groups confirmed that the two groups were similar with no significant differences in angiographic findings after recurrent angina and these treatment approaches.

Recurrent angina after CABG is always a frustration to both the patient and the physician. For most surgeons an occluded graft anastomosis is seen as a surgical fault, while an occluded native vessel counts as fate^[6]^. Most common causes of early recurrent angina are technical errors, poor target vessel runoff, graft insertion site lesion, and IR. This is an indication for prompt CAG with PCI, if feasible^[3]^. The main factors of SVG failure during the first postoperative year has been suggested by Cataldo et al.^[7]^ to be a small vessel diameter, reduced wall motion of the vessel-dependent myocardial region, and the right coronary as target vessel. In our study, 45 (35.4%) patients had recurrent angina in the first postoperative year. There were new native vessel stenosis in 18 (40%) patients and graft failure in 28 (27.7%) grafts. Recurrent angina after the first year (called late recurrent angina) can occur with the development of stenosis in a graft (either saphenous vein or arterial) or with progression of atherosclerosis in native vessels^[3]^. Late recurrence was seen in 82 (64.6%) patients with recurrent angina after the first year of CABG. Thirty-two (39%) patients had new native vessel stenosis, and graft failure was seen in 38 (20%) grafts.

The incidence of recurrent angina after CABG varies considerably among reported studies^[4,8]^. Cameron et al.^[9]^ reported that after CABG, 24% of patients had angina at the first year, which increased to > 40% by the sixth postoperative year. Early graft failure is considered to be largely dependent on procedure complications and occurs in up to 15% of cases^[10,11]^. In our study, it is difficult to say the exact incidence of recurrent angina after CABG because the study was retrospective, and it is very difficult for some patients to know when and how they have chest pain. This is unfortunately one of the major limitations of this study. However, roughly the incidence of chest pain requiring CAG is approximately 10.7%.

Patients with unstable angina generally should proceed directly to CAG, whereas patients with stable angina may be candidates for noninvasive testing to evaluate their risk and the extent of myocardial hazard^[12]^. The impact of CAG early after CABG for suspected postoperative myocardial ischemia was investigated by Rupprecht et al.^[6]^ One hundred eight patients suspected with ischemia underwent postoperative CAG after CABG. Seventy-nine (73%) patients demonstrated graft pathologies. Fifty-two (48%) of these patients were treated with PCI (stent implantation). The main inclusion criterion of this study was myocardial ischemia after CABG defined as an increase of creatine kinase-myocardial band within 48 hours after uneventful surgery. In our study, the inclusion criteria were CAG with or without PCI after CABG in patients with recurrent angina after CABG. Our study population was different in that it incorporated patients who had angina after CABG.

The finding of CAG after CABG was reported to be normal in 42 to 67% of patients. Graft pathologies were noted in 33 to 58% of cases^[13-15]^. Janiec et al.^[16]^ showed that most of the postoperative recurrence of CAD symptoms are possibly attributable to internal mammary artery (IMA) failure or progression of atherosclerosis in native coronary arteries. SVG are most often used in CABG but are exposed to graft disease^[17]^, and their reduced long-term patency is well established when compared to IMA grafts^[18]^. Sergeant et al.^[19]^ reported that recurrent angina after CABG has minimal impact on survival and is not predictive of myocardial infarct. Occurrence of chest pain in a patient with normal angiographic findings, usually associated with ST-segment depression during spontaneous or provoked angina, is called cardiac syndrome X. Angina is thought to be secondary to microvascular dysfunction^[20]^. In our study, there were 11 (24.4%) patients in group I, and 25 (30.5%) patients in group II with normal angiographic findings. These patients were treated medically. All of them had stable angina pectoris, and none of them had myocardial infarction findings. It was shown that optimal medical treatment had a bigger impact on outcomes than the choice of revascularization method^[21]^.

Several studies have shown that CAG and PCI can be performed safely after CABG^[22,23]^. Repeated CAG and the need for reintervention were not rare after CABG. The incidence of CAG and the need for intervention were higher in patients operated with only a single or no SVGs in addition to the IMA when compared to those with multiple additional SVGs^[16]^. The type of bypass conduit and risk factors for arteriosclerosis affect the need for coronary reintervention. Aggressive post-CABG risk factor reduction and maximum arterial grafting at primary operation should decrease coronary reinterventions^[24]^. Not all patients with recurrent angina and significant angiographic findings following CABG underwent further PCI. In the literature, patients treated medically only include 10 to 20% of all patients^[13-15]^. In our study, PCI is mostly favored for patients’ comfort and its technical ease in forty-nine patients. Because PCI is not always feasible, forty-two patients were conservatively treated without intervention.

### Limitations

The main limitation of this study is its retrospective and observational nature. Moreover, our study included all patients who had recurrent angina leading to CAG but did not include those patients who may have had an ischemic event but did not undergo CAG or intervention. In our study, it is difficult to say the exact incidence of recurrent angina after CABG. This is one of the major limitations of this study.

## CONCLUSION

Recurrent angina is a complaint that should not be neglected because most of the patients with recurrent angina are diagnosed with either native coronary or graft pathology in CAG. Postoperative CAG is a useful tool with a significant therapeutic value.
